# Network Analysis Shows Asymmetrical Flows within a Bird Metapopulation

**DOI:** 10.1371/journal.pone.0166701

**Published:** 2016-11-28

**Authors:** Emilio R. Rojas, Cédric Sueur, Pierre-Yves Henry, Blandine Doligez, Gérard Wey, Olivier Dehorter, Sylvie Massemin, Groupe Cigognes France

**Affiliations:** 1 IPHC, UNISTRA, CNRS, Strasbourg, France; 2 Mécanismes adaptatifs et Evolution (MECADEV UMR7179), Sorbonne Universités, MNHN, CNRS, Muséum National d'Histoire Naturelle, 1 avenue du Petit Château, Brunoy, France; 3 Centre de Recherches sur la Biologie des Populations d’Oiseaux (CRBPO), Centre d’Ecologie et des Sciences de la Conservation (CESCO UMR7204), Sorbonne Universités, MNHN, CNRS, UPMC, Muséum National d’Histoire Naturelle, Paris, France; 4 UMR CNRS 5558 –LBBE, "Biométrie et Biologie Évolutive", Villeurbanne cedex, France; 5 Groupe Cigognes France & APRECIAL, Colmar, France; University of Lleida, SPAIN

## Abstract

How the spatial expansion of a species changes at a human time scale is a process difficult to determine. We studied the dispersal pattern of the French white stork population, using a 21-year ringing/resighting dataset. We used the graph-theory to investigate the strength of links between 5 populations (North-East, North-West, Centre, West, and South) and to determine factors important for the birds’ movements. Two clusters of populations were identified within the metapopulation, with most frequent movements of individuals between North-Eastern and Centre populations, and between North-Western and Western populations. Exchanges of individuals between populations were asymmetrical, where North-Eastern and North-Western populations provided more emigrants than they received immigrants. Neither the geographical distance between populations, nor the difference in densities influenced the number of individuals exchanging between populations. The graph-theory approach provides a dynamic view of individual movements within a metapopulation and might be useful for future population studies in the context of conservation.

## Introduction

Theory of metapopulation focuses on the dynamics of individual exchanges between populations [1,2]. It is based on the accumulation of field records concerning the dispersal behaviour of individuals and/or observed gene flow between populations [3]. The majority of past studies focused on the impact of habitat fragmentation, since animal populations were patchily distributed, forming metapopulations. Another important aspect that has been studied concerns the risk of species extinction in dependence of population size and the degree of connectivity between populations. The role of individual dispersal between populations is crucial for the persistence of a metapopulation, in particular for threatened species (e.g. the Cougar, *Puma concolor* or the Florida scrub-jay, *Aphelocoma coerulescens* [4,5]). Ecological factors also need to be studied to understand the mechanisms that, for instance lead to a wider geographical distribution of a metapopulation [6]. While biogeography studied the expansion of species at the level of islands or continents and over periods of several thousand years, determining the role of individual dispersal behaviour may help to define the dynamics of metapopulations on a finer geographical and temporal scale. Harrison [7] proposed that individual qualities determined how individuals disperse. For instance, young individuals have a propensity to leave the natal site, to travel long distances over unfavourable habitat, have a higher ability to locate new habitats and, therefore, a higher capacity to establish new populations from a small numbers of founders. How the spatial expansion of a species changes at the level of a human time scale (i.e. less than 40 years) is difficult to determine. To study the intensity of individual exchanges between populations and the factors influencing dispersal patterns, we used the white stork (*Ciconia ciconia*) as our model species. Until the 1970’s, the distribution of white storks was restricted to the North-Eastern region of France (i.e. Alsace) but has expended in recent decades [8]. In 1961, the Alsatian population consisted of 145 pairs but declined to only nine pairs in 1974 [9]. Such a fast, drastic decline was also observed in other white stork populations of Western Europe [10–12]. After the implementation of several conservation programs in Switzerland and the Alsatian region, these populations increased again [8,13,14]. In France, the North-Eastern population size increased to an almost five fold level in 2015, when compared with the situation in 1961 (Groupe Cigognes France). Other populations started to establish themselves in different regions of France during the 1980’s [15], for example in Charente-Maritime [16,17] or in the North-Western regions of France [8]. Moreover, the colonizing white storks have the capacity to install in new habitats. Dispersal in juveniles is high (natal dispersal: 70% of individuals) and is also observed in adults (breeding dispersal: 6%) [16–18]. Hence, juveniles make up good candidates to disperse and found new populations. The build up of the French white stork metapopulation, after its near extinction in the 1970’s, has not been analysed yet. Information is only available for the Charente-Maritime population and only for the period between 1991 and 1996 [16]. We studied the dispersal patterns of white storks on the national scale (i.e. France) and investigated the importance of the North-Eastern population as a source population for the establishment of new populations within other regions of France. For this purpose, we used the graph-theory to study the strength and dynamics of links between populations. Graph-theory is a mathematical approach dealing with the connectivity inside a network [19]. In biology, it has been used in particularly for the analysis of social networks [20–23]. Its use widened to study landscape networks [19,24–26] and it has recently emerged as a powerful tool to study the exchanges of individuals between populations [27,28].

In our analysis, we used a ringing/resighting dataset concerning mainland France (~544,000 km^2^), covering a 21-year period (from 1988 to 2008). Such a large spatial and temporal coverage is essential to investigate the movement patterns within a metapopulation for far-ranging species, such as white storks. The main aim of our study was to investigate the potential asymmetry of individual exchanges between populations to diagnose ‘source-sink’ dynamics during the build-up of a metapopulation. We studied several factors that can affect the dispersal patterns of white storks: 1) attractive or repulsive effects of conspecifics, which have been reported in the literature [29–31]. We tested the effect of the relative density of conspecifics to investigate the trade-off between competition and gregarious behaviour [30,32]. 2) The effect of inter-population distances was evaluated, with the prediction that the majority of individuals would disperse between populations that are closer to each other. 3) The presence of population sub-groups with preferential exchanges of individuals, called clusters, was also investigated in order to assess dispersal patterns inside the metapopulation.

## Materials and Methods

### Study species and data collection

The white stork is a large bird that lives in close association with humans [33] and is present in numerous cultivated habitats in Central and Western Europe [34]. After a critical drop of the Western European stork populations in the 1970’s [11,35], conservation programs succeeded to increase bird numbers to their former levels or even beyond. In France, the current white stork population size is higher than before the drop [16,35,36].

White storks were ringed as nestlings (~1.5-month old) using uniquely marked individual rings that could be read up to a distance of 50–70 m using spotting scopes. Ringing and resighting data (i.e. individual identity, date and location) were collected from 1988 to 2008 by volunteer ornithologists and bird ringers that monitor stork populations in France (coordinated by CRBPO until 2008 [37]). Ringing effort (i.e. the proportion of hatched nestlings in a given year that were ringed) decreased relative to the observed population growth. While almost all nestlings were ringed at the beginning of the study period, this was not the case later on, because of the large number of nestlings. However, we are confident in the robustness of our conclusions relative to this drop in marking probability because while the ringing effort varied along the study period, the resighting effort remained similar for each population (i.e. almost all breeders were followed).

We defined the breeding season as the period from the latest laying date recorded for the species [38] to the earliest fledging date [12,39,40], extending from mid-April to mid-July. Immature, non-breeding individuals are known to have erratic movements during the first years of their life [41–43] and were therefore excluded from the analysis. We only considered individuals that were resighted as adults (≥ 4 years old) and for which the probability to be breeders at the time of resighting was high [12,16]. We obtained a total of 1720 dispersal events (for 1087 individuals), containing 239 dispersal events between populations and 1481 dispersal events within populations.

Dispersers were defined as breeding individuals observed at a distance greater than 5km from where they had been observed during the previous breeding season (breeding dispersal) or from their nest of birth (natal dispersal; [12,16]). This 5-km threshold distance was set according to the foraging distance of breeders during the breeding season around their nest [44,45]. Dispersal distance and direction were determined from geographical coordinates of ringing and/or resighting sites. The distance between two populations was defined as the Euclidian distance between the centroids of populations. The mean distance between the centroids of populations in our study was 351.8 ± 30.3 km, a value higher than the mean dispersing distance typically found in this species (18 ±41.7 km in Germany [46], 94 ±132.2 km in Poland [43], and 67.3 ±136.2 km in France, [18]).

We defined five geographical populations, where individuals originated from (i.e. where individuals were born and ringed). The five main populations (West, North-East, North-West, South, and Centre) contained 83% of all ringed individuals of our dataset and 86% of all dispersal events.

In our analysis, we defined 5 temporal classes, with one class spanning the period of 9 years (1988–1996; due to the low number of ringed birds) and four classes spanning a period of 3 years each (1997–1999, 2000–2002, 2003–2005, and 2006–2008). We also considered the density differences observed between populations to investigate if the number of individuals within a population influenced the movements from one population to another. As we used correlation matrices in our analysis, which required one value per pair (dyad) of populations, we used density differences as an index for the effects of conspecifics density. Density was estimated based on the counts of breeding pairs made by the French national network of storks ornithologists (“Groupe Cigognes France”). In the following, the number of individuals was divided by the surface area of the territory occupied by the population (i.e. number of individuals per 100km^2^).

### Population network indices

We calculated the following parameters for the entire observation period (21 years) and for each temporal class. Our metapopulation consisted of 5 “nodes” inside the network, the five populations. Links between these nodes represent the numbers of between-population dispersal events. Links can be oriented so as to distinguish between symmetrical and asymmetrical exchanges (for instance, source-sink dispersal) between populations. We calculated the strength of nodes as the sum of dispersal events between a given population and all others [47].

We performed a cluster analysis that indicated if and how populations were connected to each other. To this end, the 1481 dispersal events within their population were excluded from these following analyses. We extracted the maximum modularity (Max. Mod.), which is the fraction of internal connections in each cluster minus the expected fraction, if connections were distributed randomly [21], and also determined the cophenetic correlation coefficients CCC [47]. Modularity indicates to what extent a group is clustered, with a value that ranges between 0 (indicating a perfectly random distribution of exchanges) and 1 (indicating a strong hierarchical clustering [21]). A modularity greater than 0.3 is usually considered to indicate useful divisions of the data [48–50]. The CCC indicates how well the dendrogram (the graphic representation of data clustering) matches the matrix of dispersal events between populations (nodes). A CCC value greater than 0.8 indicates a good match [47,50]. A cluster out/in ratio was also calculated for populations included in clusters. For a given population, the number of interactions outside its cluster was divided by the number of interactions inside its own cluster. This ratio indicated the population role in the cluster. A low out/in ratio indicated populations that interacted mostly within their cluster, while a high out/in ratio suggested external exchanges with other clusters/outside populations.

### Statistical analyses

We tested for correlations between 1) the number of individuals dispersing between two populations and the density differences between these populations; 2) the number of individuals exchanged in relation to the distance between their centroids. We obtained a matrix for each of these parameters and correlations were conducted using a Mantel Z-test [51] with 10,000 permutations.

We further investigated the attractiveness (more individuals arrive in a population than leave) or repulsiveness of a population for dispersing events. We designed three matrices: 1) a matrix containing the number of dispersing individuals within their own populations for each temporal class; 2) a matrix containing the number of individuals arriving in each population for each temporal class; and 3) a matrix with the number of individuals leaving the populations for each temporal class. Thus, we were able to perform matrix correlations using Mantel Z-tests with 10,000 permutations, to test attractiveness or repulsiveness of each population.

The asymmetry between the number of emigrating and immigrating individuals for a given population was established by means of permutation-based chi-squared tests for asymmetry (with 10,000 permutations). Network measurements and analyses were performed using SOCPROG v2.5 [47] and R (v3.1.1; R Development Core Team) softwares. Gephi [47] was used for drawing figures. Means are presented with SE.

## Results

### Dispersal at metapopulation level

**Fig 1:**
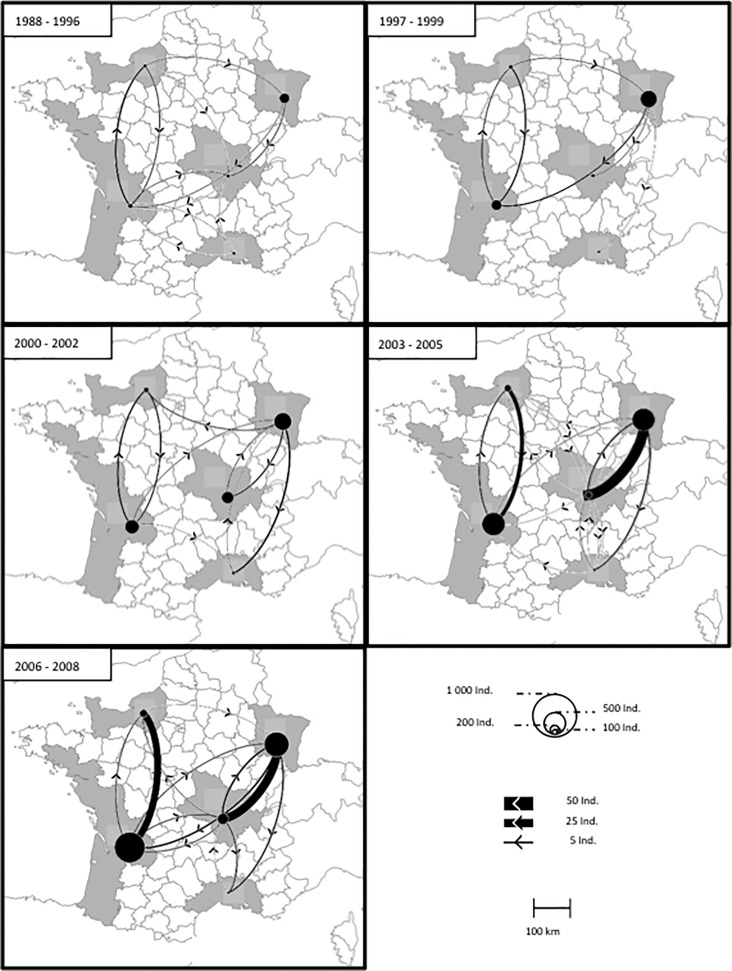
Visual representation of the metapopulation structure for white storks in France during the five periods considered. The curves thickness is proportional to the number of individuals dispersing between the populations. Each node represents one population and is positioned in its center. The size of the nodes is proportional to the number of individuals. Codes for the populations are: W for West, S for South, C for Centre, NE for North-East and NW for North-West.

The geographical distance between populations did not influence the number of individuals that they exchanged (Mantel Z-test, Z = -0.245; p = 0.851). The cluster analysis identified two distinct clusters in the global metapopulation network: cluster A, containing the North-East/Centre dyad and cluster B, containing the North-West/West dyad (Total: Max. Mod. = 0.507, CCC = 0.985; Fig 1, Table 1).

**Table 1:** Temporal evolution of the asymmetry of links, cophenetic correlation coefficients (CCC), maximum modularities (Max. Mod.), and consecutive cluster determinations for the metapopulation. Statistically significant results are presented in bold. For the asymmetry tests, “**»**” indicate the direction of the asymmetry. Codes for the populations are: W for West, S for South, C for Centre, NE for North-East and NW for North-West.

Periods	1988–1996	1997–1999	2000–2002	2003–2005	2006–2008	Total
CCC	0.948	0.954	**0.863**	**0.997**	**0.976**	**0.985**
Max. Mod.	0.272	0.285	**0.503**	**0.564**	**0.520**	**0.507**
Clusters	None	None	NE/C NW/W	NE/C NW/W	NE/C NW/W	NE/C NW/W
Asymmetry	None	None	None	NE»C NW»W	NE»C NE»SNW»W	NE»C NE»SNW»W

A detailed examination of the temporal dynamics revealed that clusters A and B only appeared during the last years considered, from 2004 onward, with a clear increase in modularity (Fig 1, Table 1). Hence, both clusters stabilised only recently. The dispersal events occurring inside (In) and outside (Out) of the clusters (out/in ratio) varied between populations (permutation-based chi-square test of asymmetry: χ^2^ = 13.94, p < 0.01; Table 2), regardless of the temporal class considered. Individuals from the Centre population in Cluster A and from the Western population in Cluster B dispersed in equal proportions in and out of the clusters. On the other hand, the North-Eastern population in Cluster A and the North-Western population in Cluster B contained mainly individuals that dispersed within their respective clusters (i.e. within-cluster dispersal). In other words, North-Eastern and North-Western populations mainly provided individuals to the other population of their cluster (respectively Centre and West populations), whereas these last populations contributed with relatively more between-cluster exchanges of individuals (i.e. they equally provided individuals to their cluster as to the other cluster).

**Table 2:** Number of dispersal events (links) occurring inside (In) and outside (Out) of the clusters for each population and the corresponding ratio between them, “Out/In”. Codes for the populations are: W for West, C for Centre, NE for North-East and NW for North-West.

Clusters	Populations	In	Out	Out/In
Cluster A	NE	78	34	0.436
	C	13	12	0.923
Cluster B	NW	52	8	0.159
	W	20	14	0.700

Dispersal links between populations (i.e. edges of the network) were asymmetrical, particularly between the North-Eastern and Centre populations (permutation-based chi-square test of asymmetry: χ^2^ = 46.43, p < 0.01), the North-Eastern and Southern populations (χ^2^ = 11.84, p < 0.01), and, finally, between the North-Western and Western populations (χ^2^ = 14.22, p < 0.01; Fig 1). This asymmetry appeared to be time-dependant and no evidence for asymmetry was found before 2003. Between 2003 and 2005, asymmetry existed only between the North-Eastern and Centre populations (χ^2^ = 25.33, p < 0.01) and the North-Western and Western populations (χ^2^ = 6.37, p = 0.01; Table 1; the former providing more dispersers than the latter). In the most recent years, between 2006 and 2008, the pattern of asymmetry remained between the North-Eastern and Centre populations (χ^2^ = 16.89, p < 0.01), and between the North-Western and Western populations (χ^2^ = 17.29, p < 0.01), but also became significant between the North-Eastern and the Southern populations (χ^2^ = 5.00, p = 0.03; Fig 1). This suggests that an asymmetry of the edges in our metapopulation appeared only very recently. In addition, we calculated nodal indices to test the strength of links between populations or nodes for each temporal class. This showed that the strength of nodes increased with time, and the strongest nodes were observed during the most recent years for the North-Eastern and Centre populations (permutation-based F-test, 10,000 permutations: F = 32.2, p = 0.030, r^2^ = 0.829; Fig 1).

### Dispersal at population level

The North-Eastern population was always larger than the Centre population, regardless of the period considered (Fig 1), especially when the two clusters were significantly different (mean ±SE number of individuals/year: 283±8 vs. 25±10 for the North-Eastern and Centre population, respectively in 2000–2002; 390±21 vs. 78±5 in 2003–2005, and 444±27 vs. 115±18 in 2006–2008). The North-Western population, on the other hand, was always smaller than the Western population (48±6 vs 218±63 for the North-Western and Western population, respectively in 2000–2002, 89±8 vs. 397±63 in 2003–2005, and 120±11 vs. 553±57 in 2006–2008).

The difference in densities did not influence the number of individuals exchanging between populations (Mantel Z-test, Z = 0.188; p = 0.250). However, the number of dispersing events within a population can influence its attractiveness/repulsiveness. We found no evidence for an attractiveness effect (Mantel Z-test, Z = 0.197; p = 0.208), while we did find significant evidence for a repulsiveness effect (Mantel Z-test, Z = 0.871; p = 0.008). This indicates that a high level of internal dispersal activity leads to more individuals leaving the population, thus, increasing the contribution of the population to metapopulation dynamics.

## Discussion

We conducted a network analysis concerning the strength of links between 5 populations within a white stork metapopulation that increased in number of individuals between 1988 and 2008. We found that the metapopulation is divided into two clusters, opposing North-Eastern/Centre populations to North-Western/Western populations. Clustering was detected starting in 2000, whereas the establishment of a metapopulation was already observed at the beginning of the study period (i.e. 1988). Clustering seems to be sensible to an increasing number of dispersing events, associated with an increase of the different white stork populations in France [16,35,36]. From 1999 onward, the number of dispersal events between populations further increased. The asymmetry analysis conducted in our study suggests a source-sink relationship between populations [52]. Thus, emigration from the North-Eastern and North-Western populations was greater than immigration, so that these populations, took on the role of “source” populations, while the other populations, Western, Centre, and Southern, were “receiver” populations, this immigration likely contributing to their maintenance or growth. However, while the North-Eastern population is larger than the Centre population, the North-Western population is smaller than the Western population. Consequently, there is no support for a density effect that has been observed in Polish and German white stork populations [16,17]. In Germany, the study of Itonaga and colleagues showed that the proportion of dispersers in white storks increased in parallel with the population size [46]. These findings support indirectly the influence of density on the dispersal rate. There are several explanations for this seemingly contradictory pattern of density-dependence in white storks. First, the U-shaped relationship between density and dispersal distance might be responsible for the observed pattern [53]. In colonial species, the dispersal probability/distance is high in populations with a moderate to low density (with birds moving to higher density populations, cf. Allee effect) and also in populations with a moderate to high density (with birds moving to lower density populations, cf. negative density-dependence effect). Consequently, the dispersal probability could be low in the populations with a moderate density, underlining the U-shaped relationship. Secondly, the observed dispersal rate might not depend so much on the distance or the proximity between two populations but rather might be linked with the migratory behaviour of this species. Thus, the pattern observed in in our study, contradictory to previous studies, might largely depend on the migratory axis, which could potentially mask a density effect. White storks coming back from their wintering grounds disperse and settle along the North/South migration axis [16,18]. This might explain why the Northern “source” populations (North-East and North-West) provided more individuals than they received, as the asymmetry and cluster out/in analyses have shown. However, the Western population cannot be considered as a sink population, since its population size increased at a higher rate than the North-Western population, and its population increase might be more likely related to high breeding success and habitat quality. In future analysis, a more biologically realistic picture of the ‘French’ white stork metapopulation dynamics should be obtained by taking into account international dispersal events: the North-Western population indeed largely extends in western Germany [9], the Eastern cluster is also connected to the reintroduced Swiss population [54], whereas the Southern and Western populations have exchanged some individuals with Spanish populations [17]. Finally, we have shown that the dispersal within a population can have an apparent repulsive effect. Populations with a high internal dispersal were also the ones also had the highest rate of between-populations dispersal. This suggests that the factors influencing dispersal initiation (departure), either within or between populations, most likely are the same [55]. We did not find a density dependence of dispersal events, but one should keep in mind that cost/benefit trade-offs for dispersal are influenced by different factors, not all tested in our study. These factors could be abiotic, such as climate variation [56,57], biotic, such as density, predation pressure or prey distribution [30,53,58], or genetic, like kin competition and inbreeding avoidance [3,59]. Interplay of all these factors can directly affect the metapopulation dynamics [31,55], especially the source/sink role of populations.

In conclusion, the aim of our study was to highlight the asymmetric contribution of different populations in the buildup of a metapopulation, after near extinction. Few studies have simultaneously considered dispersal movements by using gene flow and marked individuals [27,60] that reflect past and more recent dispersal between patches/populations [5]. It was possible with our time-series database to consider the dispersal behavior at both levels (intra- and interpopulational) and thus built the metapopulation network for the different time periods. For conservation purposes, this type of analysis is extremely useful, as it allows identifying the sources from which individuals emigrate to found and build-up new populations. It focuses on populations that receive less and less individuals, requiring special attention in order to guarantee the existence of endangered species populations.

## Supporting Information

S1 Dataset Supporting information-metapopulation-white storks.xlsx” (XLSX)
